# Low-dose Bisphenol-A Promotes Epigenetic Changes at *Pparγ* Promoter in Adipose Precursor Cells

**DOI:** 10.3390/nu12113498

**Published:** 2020-11-13

**Authors:** Michele Longo, Federica Zatterale, Jamal Naderi, Cecilia Nigro, Francesco Oriente, Pietro Formisano, Claudia Miele, Francesco Beguinot

**Affiliations:** 1 URT Genomics of Diabetes, Institute of Experimental Endocrinology and Oncology, National Research Council, 80131 Naples, Italy; mi_longo@libero.it (M.L.); federicazatterale@libero.it (F.Z.); jamal.naderi83@gmail.com (J.N.); c.nigro@ieos.cnr.it (C.N.); foriente@unina.it (F.O.); fpietro@unina.it (P.F.); c.miele@ieos.cnr.it (C.M.); 2 Department of Translational Medicine, Federico II University of Naples, 80131 Naples, Italy

**Keywords:** obesity, Bisphenol-A, food contaminant, environmental pollutant, adipogenesis, adipose tissue, insulin resistance, diabetes, inflammation, adipose tissue dysfunction

## Abstract

Exposure to endocrine-disrupting chemicals such as Bisphenol-A (BPA) is associated with an increase in obesity prevalence. Diet is the primary cause of human exposure to this contaminant. BPA promotes obesity by inducing adipocyte dysfunction and altering adipogenesis. Contradictory evidence and unanswered questions are reported in the literature concerning the BPA effects on adipogenesis. To clarify this issue, we tested the effects of prolonged low-dose BPA exposure on different phases of adipogenesis in committed *3T3L1* and uncommitted *NIH3T3* preadipocytes. Our findings show that BPA effects on the adipogenesis are mediated by epigenetic mechanisms by reducing peroxisome proliferator-activated receptor gamma (*Pparγ*) promoter methylation in preadipocytes. Nevertheless, in BPA-exposed *3T3L1*, *Pparγ* expression only transiently increases as lipid accumulation at day 4 of differentiation, without altering the adipogenic potential of the precursor cells. In the absence of differentiation mix, BPA does not make the *3T3L1* an in vitro model of spontaneous adipogenesis and the effects on the *Pparγ* expression are still limited at day 4 of differentiation. Furthermore, BPA exposure does not commit the *NIH3T3* to the adipocyte lineage, although *Pparγ* overexpression is more evident both in preadipocytes and during the adipocyte differentiation. Interestingly, termination of the BPA exposure restores the *Pparγ* promoter methylation and inflammatory profile of the *3T3L1* cells. This study shows that BPA induces epigenetic changes in a key adipogenic gene. These modifications are reversible and do not affect preadipocyte commitment and/or differentiation. We identify an alternative transcriptional mechanism by which BPA affects gene expression and demonstrate how the challenge of preventing exposure is fundamental for human health.

## 1. Introduction

In the last decade, the prevalence of obesity has risen significantly, but despite considerable attempts to establish the underlying mechanisms, the causes of the epidemic remain unclear [1,2]. Importantly, type 2 diabetes, metabolic syndrome, cardiovascular disease, and cancer are also associated with obesity prevalence [3,4,5].

The causes of the alarming rise in the incidence of these diseases include excessive calorie consumption, food composition, physical inactivity, and exposure to environmental pollutants such as endocrine-disrupting chemicals (EDCs) [6]. Some EDCs are considered obesogenic and associated with the etiology of obesity [6,7,8,9] and diabetes [10,11]. According to the definition of the International Programme on Chemical Safety (IPCS) of the World Health Organization (WHO), EDCs are exogenous substances or mixtures that alter the functions of the endocrine system and consequently cause adverse health effects in an intact organism, or its progeny [12]. Such chemicals are ubiquitous and extensively contaminate food and water [12,13]. Industrial food processing unintentionally enables these pollutants to enter the food chain and accumulate in wildlife and humans. Approximately 800 chemicals are assumed to interfere with endocrine functions, including BPA and phthalates. The primary source of these pollutants is mainly plastic food storage items, such as bottles and containers. Although these pollutants are released even at room temperature, cooling and heating strongly favor their leakage from containers, resulting in contamination of food and drink [14,15]. The inner layer of cans (made of epoxy resins) is another big source of BPA. As a result, products in bottles and plastic containers have the highest concentrations of BPA independently of the specific nutrient category [16,17,18].

In accordance with the “obesogen hypothesis”, EDCs may contribute to the epidemic of obesity and its related disorders [1,19,20]. EDCs promote weight gain directly by causing adipocyte hypertrophy and/or hyperplasia [1,19] or indirectly by altering the metabolism and hormonal regulation of appetite and satiety [19,20,21,22,23].

Among EDCs, BPA, the most studied member of these compounds, is a small molecule commonly used in the manufacture of polycarbonate, epoxy, and other polymer compounds [24,25]. BPA is used in food and beverage packaging, dental sealants, and several other consumer products. In humans, it can be detected in cord serum, breast milk, placenta, urine, and blood at biologically relevant doses [26,27,28,29,30,31,32,33,34,35].

BPA is a xenoestrogen that works through several receptor-pathways to disrupt the normal functioning of the endocrine system [24,36]. BPA mimics natural estrogen 17-β-estradiol by activating alpha and beta estrogen receptors (ERs). Its affinity is over 1000–10,000 times lower than that of 17-β-estradiol for both ERα and ERβ [36,37,38]; for this reason, it is considered a very weak environmental estrogen. However, circulating concentrations of BPA fall beyond the biologically active range [39]. Besides its estrogen activity, BPA exhibits anti-thyroid [40] and anti-androgen activity [41] as well as glucocorticoid receptor stimulation [42,43]. Further studies have also proved that low (pico and nanomolar) concentrations of BPA have multiple effects on cellular physiological functions [36,38].

Human exposure to BPA occurs for both dietary (drinking water and food) and non-dietary sources (such as dust, air pollution, and thermal paper) [44,45,46]. BPA exposure studies have shown that diet is the primary cause of human exposure to this contaminant. This is primarily attributable to the transfer of BPA from food contact materials (such as polycarbonate and epoxy resins) to food and drinking water [44,47,48,49,50]. Dietary exposure is the predominant source of BPA in the general population, exposure from non-food sources is generally lower by at least one order of magnitude and associated with an occupational risk. [50].

Numerous epidemiological studies have shown the obesogenic effect of BPA. Elevated levels of BPA in biological fluids, such as urine, are associated with a higher risk of obesity [8,51,52,53]. The most compelling evidence of the BPA effect is obtained from in vitro and in vivo studies. Such findings indicate that BPA promotes adipocyte dysfunction and affects adipogenesis, although, in the latter case, the data in literature appear to be somewhat discordant. Studies conducted in vitro and ex vivo (in murine *3T3L1*, *CH310T1/2* cells, and human adipose-derived stromal cells) as well as in *C57BL/6J* mice have shown that exposure to BPA causes adipocyte dysfunction as it induces a decline in insulin sensitivity and glucose tolerance for the inhibitory effects on insulin signaling [25,39,54,55]. Besides that, BPA has also shown to promote the expression and release of certain proinflammatory cytokines, resulting in a low-grade inflammatory state at local and systemic levels [25,56]. In our previous studies on murine and human preadipocyte cells, we observed that prolonged exposure to 1 nM of BPA impaired insulin signaling, decreased insulin-stimulated glucose utilization, and increased proinflammatory cytokine expression and release [57,58]. These findings support the hypothesis that exposure to BPA leads to metabolic dysfunction and inflammation of the adipocytes, raising the risk of developing obesity and its related metabolic disorders [59,60]. Although the data in literature agree on the harmful effects of BPA on adipocyte function, the same cannot be said for the BPA effects on adipogenesis. In vitro investigations in murine and human cells showed that exposure to high (1–50 μM) and low range of BPA doses (10 nM–1 μM) during adipocyte differentiation led to higher lipid accumulation in adipocytes. The expression of adipogenic marker genes such as *Pparγ*, the key transcription factor involved in adipogenesis, is reported to be increased when tested [61,62,63]. Prenatal BPA exposure (pregnant rats exposed to BPA doses of 1 mg/L in drinking water) leads to a significant increase in the birth weight of both the male and female offspring. This excess of white adipose tissue was associated with adipocyte hypertrophy and enhanced expression of pro-adipogenic genes such as *Pparγ* and lipoprotein lipase [64]. On the other hand, other studies have reported that exposure of murine and human cells to high (80 μm) and low (0.01–1000 nM) BPA concentrations for short and long periods does not increase the expression of *Pparγ* and other adipogenesis-associated marker genes and adipocyte lipid accumulation [25,61,65,66,67]. Such discrepancies in findings can be explained by a multitude of factors, such as the use of more modern methods for the assessment of adipogenesis, the cellular models used for the analysis (data in human cell lines are more discordant), the very high doses of BPA used, the exposure time, and the treatment window adopted.

Furthermore, studies investigating the relationship between the exposure to environmental chemicals and adipogenesis demonstrated the impact of EDCs on epigenetic processes. These provided the first evidence of how the DNA methylation profile of adipogenesis-related genes could be altered by the EDCs exposure [68]. We have also reported how the expression of several genes, fundamental for adipocyte differentiation, is regulated by epigenetic mechanisms [69,70,71]. Human preadipocytes give rise to adipocyte cells through a highly orchestrated program called adipogenesis. This program is not only influenced by genetic factors, but also by epigenetic factors [72] that contribute to the establishment of an exclusive gene expression program. Epigenetic silencing in mesenchymal stem cells is also fundamental in the repression of genes for alternative lineages determination. In humans, subcutaneous adipose tissue (SAT) hypertrophy appears to be a consequence of impaired adipocyte precursor cell differentiation into mature adipocytes. Alterations in epigenetic mechanisms limit the expandability and recruitment of new cells in SAT, leading to prominent adipocyte hypertrophy, which is associated with ectopic accumulation of fat, functional dysregulation of SAT, low-grade chronic inflammation, decreased insulin sensitivity, and enhanced oxidative stress [60,69,70,73].

To shed light on the matter, our study aims to investigate the effects of prolonged exposure to low BPA concentrations (to mimic human exposure) on the different phases of adipogenesis and *Pparγ* expression, considering both the commitment and terminal differentiation phases. Several studies conducted in humans of different countries and ages have measured the BPA concentrations (biologically active form) in human serum. Using different analytical approaches (mass spectrometry, high-performance liquid chromatography, and ELISA) BPA concentrations in serum vary from 0.2 to 20 ng/mL [26,74]. In our study, we tested the lowest BPA concentration at which human exposure occurs (0.2 ng/mL corresponds to ~ 1 nM). To address the issue, we used two cell lines: the *3T3L1* and the *NIH3T3*, which are respectively committed and uncommitted towards the adipocyte lineage. The effect of BPA on fibroblasts, not committed to the adipocyte lineage, has never been tested before. We investigated the effect of BPA exposure termination, to test whether the induced alterations can be reversible. Since the mechanisms by which BPA exerts its effects on *Pparγ* expression have not yet been fully recognized, we speculate that these effects could be (partially) mediated by epigenetic mechanisms.

## 2. Materials and Methods

### 2.1. Materials

Media, sera, insulin, antibiotics, and SuperScript III were obtained from Invitrogen (San Diego, CA, USA). BPA, 3–isobutyl–1–methylxanthine (IBMX), rosiglitazone, and dexamethasone (DEX) were purchased from Sigma-Aldrich (St. Louis, MO, USA). iQ SYBR Green Supermix was from Bio-Rad (Hercules, CA, USA), and EZ DNA Methylation Kit was from Zymo Research (Orange, CA, USA). DNA Purification Kit and pGEM-T Easy Vector Systems were obtained from Promega (Madison, WI, USA). QIAzol, QIAprep Spin Miniprep Kit, miRNeasy mini kit, miScript II RT Kit, miScript SYBR Green PCR Kit, and the PCR Purification kit were purchased from QIAGEN (Hilden, Germany). Big Dye Terminator v3.1 Cycle Sequencing Kit was obtained from Applied Biosystems (Foster City, CA, USA). MTT assay kit was purchased from Biotium, Inc. (Hayward, CA, USA).

### 2.2. Methods

#### 2.2.1. Cell Culture and Adipocyte Differentiation

*3T3L1* and *NIH3T3* mouse embryonic fibroblasts were purchased from the American Type Culture Collection (Manassas, VA, USA). Both cell lines were mycoplasma-free and cultured in Dulbecco’s modified Eagle medium (DMEM) (Invitrogen) complemented by 10% calf serum (CS), penicillin (200 IU/mL), and streptomycin (100 g/mL). The cells were maintained at 37 °C in a humidified incubator with 5% CO_2_. The *3T3L1* and *NIH3T3* cells were cultured in the presence of BPA (1 nM) and vehicle (methanol) for 8 days before the induction of adipogenesis and during the differentiation process. Cells were subcultured in T75 culture flasks for maintenance when the confluence reached about 70%. All experiments were performed in cells between passages 4–6. *3T3L1* and *NIH3T3* cells were seeded at a density of 6 × 10^4^ cells per well and cultured in 3 mL of medium. *3T3L1* and *NIH3T3* cells were treated with BPA (1 nM) or vehicle (methanol) for 8 days before the induction of adipogenesis and during the differentiation process. The medium containing the vehicle or BPA was replaced every other day during both the pretreatment and the adipocyte differentiation phases.

For adipocyte differentiation, cells were grown to confluence in 10% CS medium. After 2 days of confluence, adipocyte differentiation was induced using DMEM 10% of fetal bovine serum (FBS) supplemented with a differentiation mix (5 μg/mL of insulin, 0.5 mmol/l of IBMX, 1 mol/l of DEX, and 1 μM of rosiglitazone). Forty-eight hours later, cells were maintained in DMEM 10% FBS and 5 μg/mL insulin, for additional 6 days. The medium was replaced every other day [75]. During the adipocyte differentiation experiments, BPA and methanol were replaced every time the adipogenic mix was changed.

For spontaneous adipocyte differentiation experiments, cells were cultured in DMEM 10% CS. After forty-eight hours of confluence, DMEM 10% CS was replaced with DMEM 10% FBS without the addition of differentiation mix. The culture medium was replaced every other day and cells were kept in DMEM 10% FBS for the following 6 days of the differentiation process. Lipid accumulation of mature adipocytes was determined by Oil red O staining. The cells were fixed in 4% formaldehyde and incubated for 20 min at room temperature. The formaldehyde was removed by washing the samples for 5 min in PBS and the cells were incubated for 60 min at room temperature in the Oil Red O staining solution. Images were taken using an Olympus microscope system (Olympus, Center Valley, PA, USA). For quantification, absorbance was measured at 490 nm using a spectrophotometer (Beckman, Los Angeles, CA, USA), after the addition of isopropanol and the incubation at room temperature for 10 min. A schematic flowchart of the experimental design is reported in the Supplementary Figure S1.

*3T3L1* cells (10^4^ cells per well) were plated in 96-well plates. At the end of the pretreatment, cell viability was measured using an MTT assay kit (Biotium, Inc. Hayward, CA, USA) according to the manufacturer’s instructions.

#### 2.2.2. RNA Isolation and Quantitative Real-Time PCR

Total RNA was extracted from *3T3L1* and *NIH3T3* cells using QIAzol reagent (Qiagen) according to the manufacturer’s protocols. One thousand RNA nanograms were retro-transcribed using SuperScript III Reverse Transcriptase (Life Technologies, San Diego, CA, USA). The cDNA obtained was used as a template for quantitative real-time PCR (qPCR), performed in triplicate using iQ SYBR Green Supermix on an iCycler real-time detection system (Bio-Rad, Hercules, CA, USA). Relative quantification of gene expression was relative to the control (equal to 1) and was calculated according to the comparative 2^−ΔΔCt^ method based on the cycle threshold (Ct) values of the target and housekeeping genes. Data normalization was achieved using the housekeeping Cyclophilin A gene as internal control. For each sample, the Ct value of each target gene was measured and compared to Ct value of housekeeping gene as ΔCt, (ΔCt = Ct target gene−Ct housekeeping gene). The fold change of target genes in experimental samples relative to control samples was determined by 2^−ΔΔCT^, where ΔΔCt = ΔCt experimental sample −ΔCt control sample. The primer sequences used for the gene expression analysis are listed in Table 1.

#### 2.2.3. DNA Methylation Assessment

Genomic DNA was extracted using a DNA Purification Kit (Promega, Madison, WI, USA). Bisulfite conversion of genomic DNA was performed using the EZ DNA Methylation Kit (Zymo Research, Orange, CA, USA). The bisulfite-converted genome was amplified by PCR using bisulfite-specific primers for *Pparγ* promoter (see Table 1 for primer sequences). For bisulfite sequencing, each PCR amplicon was subcloned using pGEM-T Easy Vector Systems (Promega, Madison, WI, USA). Then, competent *Escherichia coli* cells were transformed and plated on X-GAL (5-bromo-4-chloro-3-indolyl-beta-D-galacto-pyranoside) / IPTG (isopropyl-β-D-thiogalactopyranoside) LB (Luria-Bertani broth)-ampicillin Agar plates for screening selection. Bisulfite genomic sequencing was performed as previously reported [76]. DNA sequencing was performed on an ABI 3500 Automatic Sequencer using Big Dye Terminator v3.1 (Applied Biosystems, Foster City, CA, USA). All procedures were performed according to the manufacturer’s instructions.

#### 2.2.4. Reverse Transcription and Quantification of MicroRNAs Expression

Several algorithms for predicting microRNAs target genes are available online. These tools provide a complete overview of the predicted (with a score) and validated targets for the given microRNA and theoretical match locations for the target sequences. The following computational tools miRDB (http:/mirdb.org/) and TargetScan (http:/www.targetscan.org/archi-ves.html) were used to identify microRNAs targeting the 3′UTR of the *Pparγ* gene. The analysis was carried out using the default parameters of the above-mentioned computational algorithms. All 27 identified microRNAs are reported in Supplementary Table S1. Total RNA was isolated from BPA and vehicle-treated *3T3L1* and *NIH3T3* cells using the miRNeasy mini kit (QIAGEN, Hilden, Germany), according to the manufacturer’s instructions. Total RNA was reverse transcribed using the miScript II RT Kit (QIAGEN, Hilden, Germany), and the differential expression of microRNA 27a and microRNA 27b was analyzed by qPCR, using the miScript SYBR Green PCR Kit (QIAGEN, Hilden, Germany) and quantified as expression units relative to U6 snRNA, used as housekeeping small RNA.

#### 2.2.5. Statistical analysis

Results are shown as mean ± SD. Values for *p* between datasets were determined by two-tailed, unpaired Student’s *t*-test. Significant *p* values are indicated as *** *p* < 0.001, ** *p* < 0.01 and * *p* < 0.05.

## 3. Results

### 3.1. BPA Exerts Epigenetic Modification at Pparγ Promoter

**Figure 1:**
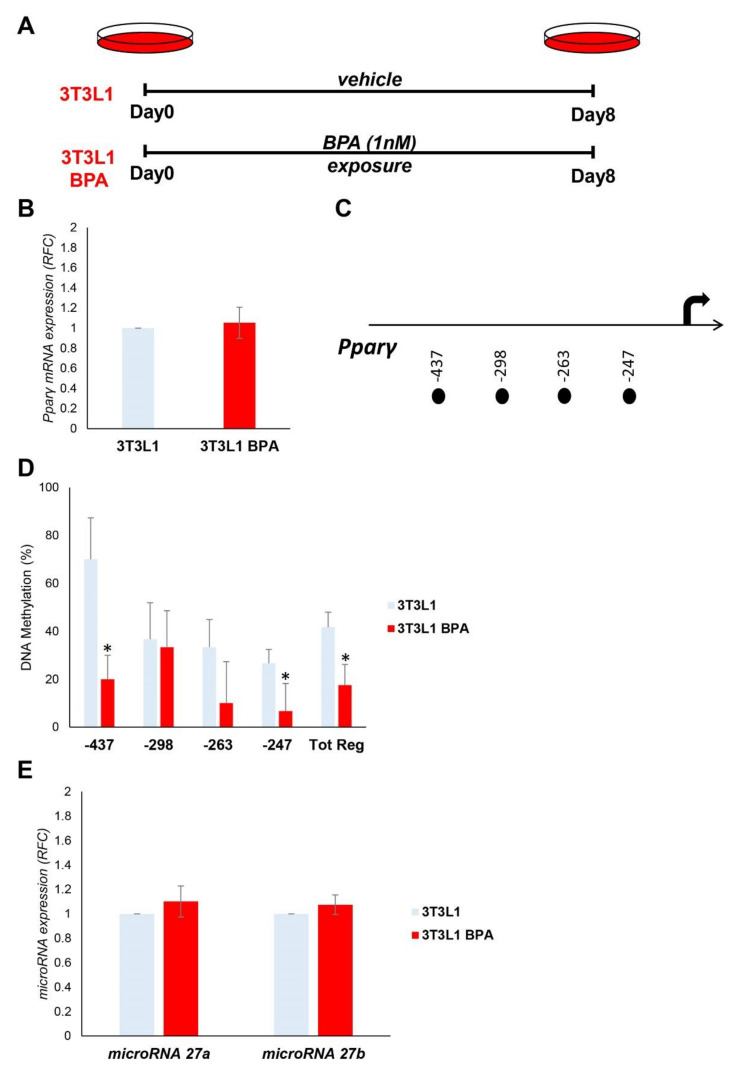
BPA exposure effects on the expression and epigenetic modifications of the *Pparγ* gene in *3T3L1* preadipocytes. (**A**) Graphical representation of the *3T3L1* cells chronically exposed (8 days) to low BPA concentrations (1 nM). (**B**) *Pparγ* mRNA levels were assessed in *3T3L1* cells exposed to BPA by qPCR. Results are the mean ± SD (*n* = 6). (**C**) Schematic representation of the four CpG sites located within the 500 bp upstream of the transcription start site (TSS) of the *Pparγ* gene, where the methylation was investigated. (**D**) Bisulfite sequencing analysis for DNA methylation assessment of the *Pparγ* promoter region in *3T3L1* cells exposed or not to BPA. Results are the mean ± SD (*n* = 3); for each experiment, at least ten clones were analyzed by Sanger sequencing. (**E**) BPA effects on the microRNA 27a and microRNA 27b expression in *3T3L1* cells. MicroRNA 27a and microRNA 27b expression was analyzed by qPCR and quantified as expression units relative to U6 snRNA used as housekeeping small RNA. Results are the mean ± SD (*n* = 5). Statistical significance was tested by the two-tailed Student’s *t*-test. Significant *p* values are indicated as * *p* < 0.05.

We tested the effect of BPA exposure on *Pparγ* expression in murine *3T3L1* preadipocytes. The *3T3L1* cells were treated with a low dose of BPA (1 nM) for 8 days (Figure 1A). Chronic exposure did not show any toxic effects in cell viability or morphology (Supplementary Figure S2). The mRNA expression of *Pparγ* in preadipocytes, measured by qPCR, was not modified by BPA treatment (Figure 1B). Nonetheless, we tested the effects of BPA on epigenetic mechanisms, which control *Pparγ* expression and are potentially established before alteration in the mRNA expression. Specifically, we measured DNA methylation at four CpG sites within 500 bp (Figure 1C) upstream the transcription start site (TSS) of *Pparγ* gene (Figure 1D). The DNA methylation of the entire region (Tot. Reg.) of the *Pparγ* promoter is reduced in *3T3L1* cells exposed to BPA (Tot. Reg. CpG methylation %: 41.7 (*3T3L1*) versus 17.5 (*3T3L1* BPA), *p* < 0.05). BPA exposure effects at 0.5 μM and 0.1 nM were also tested on *Pparγ* promoter methylation (Supplementary Figure S3).

Furthermore, we tested the expression of microRNA 27a and microRNA 27b, which have *Pparγ* as a target. As shown in Figure 1E, the expression of neither microRNA 27a nor microRNA 27b was modulated by BPA treatment in our experimental conditions.

### 3.2. DNA Hypomethylation Precedes the Increase in Pparγ Expression during the Adipocyte Differentiation

**Figure 2:**
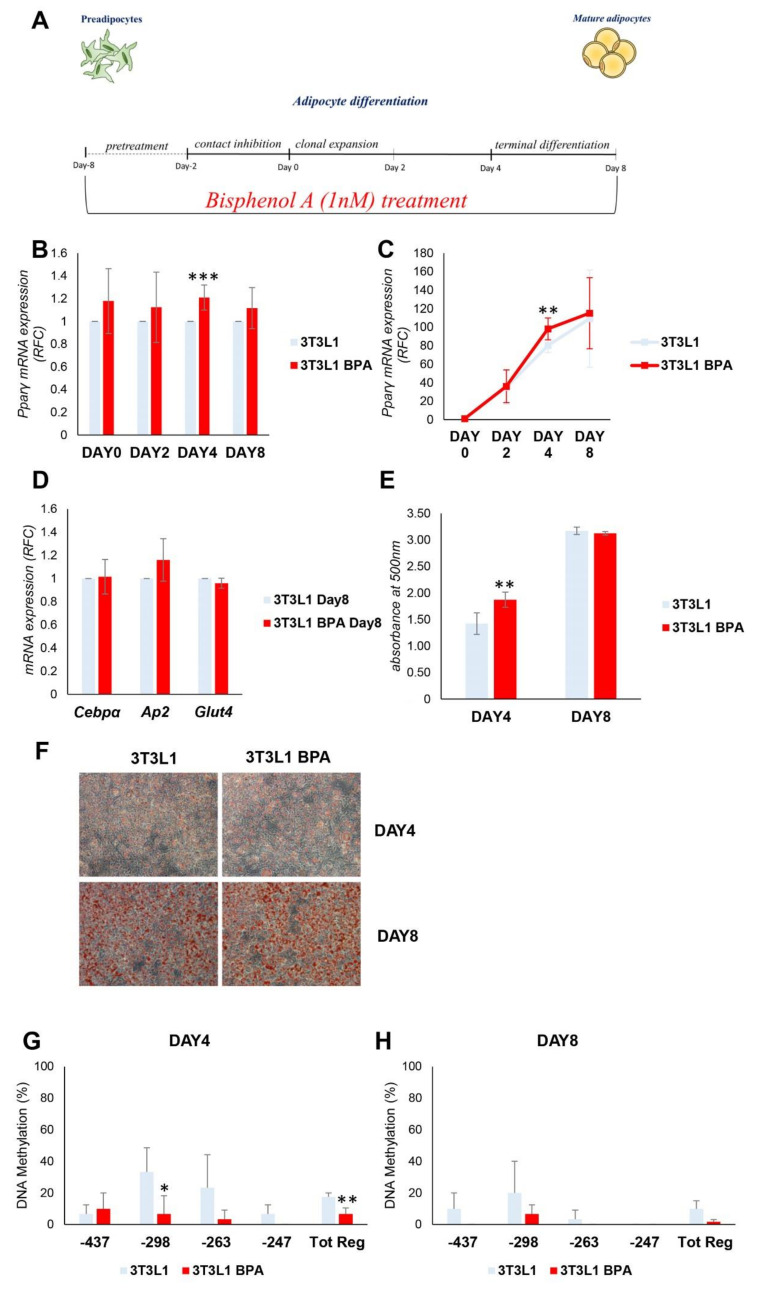
BPA exposure effects on adipogenesis of *3T3L1* preadipocytes. (**A**) A schematic representation of the *3T3L1* cells pretreated at 1 nM BPA or vehicle for 8 days. Adipocyte differentiation was induced at D0 through the administration of specific adipogenic mix. BPA or vehicle was added during the pretreatment and the adipocytes differentiation process. (**B**,**C**) *Pparγ* mRNA levels were assessed during adipocyte differentiation by qPCR. Results are the mean ± SD (*n* = 4). (**D**) The mRNA expression levels of *Cebpα*, *Ap2*, and *Glut4* were determined at D8 of adipocytes differentiation by qPCR. Results are the mean ± SD (*n* = 3). (**E**,**F**) *3T3L1* cells were fixed and stained with Oil Red O at D4 and D8 of adipocyte differentiation. (**E**) Quantification of Oil Red O staining. Results are the mean ± SD (*n* = 6). (**F**) Representative microphotographs of the staining are shown at 10×magnification; scale bars, 50 μm. (**G**,**H**) Bisulfite sequencing analysis for DNA methylation assessment of the *Pparγ* promoter region at D4 and D8 of adipocyte differentiation. Results are the mean ± SD (*n* = 3); for each experiment, at least ten clones were analyzed by Sanger sequencing. Statistical significance was tested by the two-tailed Student’s *t*-test. Significant *p* values are indicated as *** *p* < 0.001, ** *p* < 0.01 and * *p* < 0.05.

The functional impact of the above-described epigenetic changes on adipocyte differentiation was evaluated. During the adipocyte differentiation, *Pparγ* expression is strongly induced, thus we tested the BPA effect on its expression during the adipogenesis. After BPA pretreatment, adipocyte differentiation was induced in *3T3L1* cells with the differentiation mix (Figure 2A). During the process, DNA and mRNA were collected at day (D) 0, 2, 4, and 8. D8 represents the terminally differentiated adipocytes. As shown in Figure 2B,C, BPA exposure transiently and significantly increased *Pparγ* expression (1.2-fold increase) at D4 of differentiation (*p* < 0.001, *p* < 0.01). Meanwhile, no differences in mRNA expression were detected at D0 and D2. Moreover, in terminally differentiated adipocytes (D8), *Pparγ* expression was similar in BPA treated and control cells. The terminal adipocyte differentiation was assessed by the evaluation of CCAAT/enhancer binding protein alpha (*Cebpα*), fatty acid binding protein 4 (*Fabp4* or *Ap2*), and glucose transporter member 4 (*Glut4*) mRNA expression (Figure 2D). The expression of all genes analyzed does not show any differences between BPA exposed and control cells. Adipocyte differentiation of *3T3L1* cells was also assessed by evaluating the lipid content by Oil Red O staining at D4 and D8 of differentiation (Figure 2E,F). While there were no differences in terms of lipid accumulation at D8, at D4, BPA exposed cells exhibited an enhanced ability in lipid droplet accumulation (1.3-fold increase, *p* < 0.01), as highlighted by the quantification of Oil Red O staining (Figure 2E). Hence, the transient increase in *Pparγ* expression at D4 was accompanied by the increase in lipid droplets accumulation.

Following the induction of differentiation, the methylation status of CpG island on *Pparγ* promoter was then analyzed, by sodium bisulfite sequencing, at D4 and D8 (Figure 2G,H). The DNA methylation status of *Pparγ* (Tot. Reg. CpG methylation %: 17.5 (*3T3L1*) versus 5 (*3T3L1* BPA)) promoter was lower in BPA treated 3T3L1 compared to control cells at D4 (*p* < 0.01). At D8, the *Pparγ* promoter was almost demethylated both in the BPA treated *3T3L1* and in control cells (Tot. Reg. CpG methylation %: 8.3 (*3T3L1*) versus 1.6 (*3T3L1* BPA)). This suggests that the epigenetic modification precedes the increase in *Pparγ* expression in BPA exposed cells. However, the increase in *Pparγ* gene expression was transient (D4) and not associated with an improved adipogenic ability of the exposed preadipocytes.

### 3.3. Spontaneous 3T3L1 Cells Differentiation Is Not Enhanced by BPA Pre-exposure

**Figure 3:**
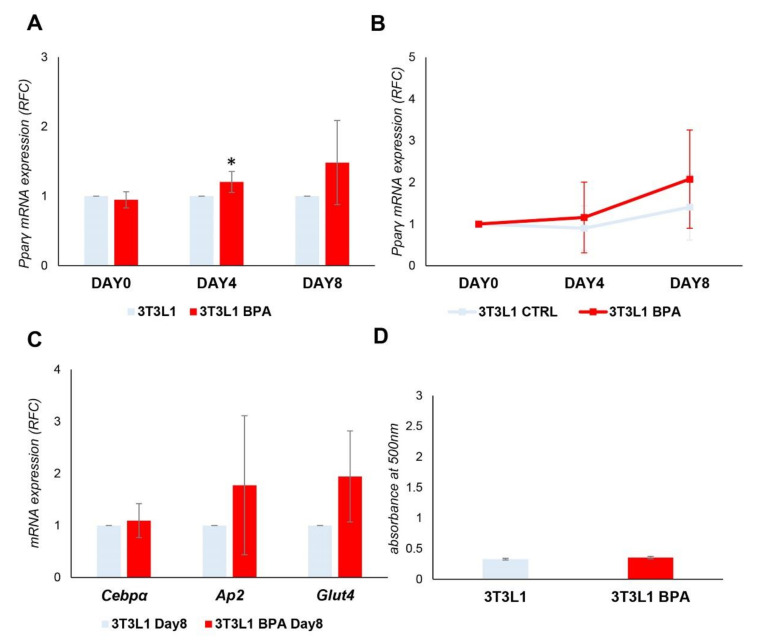
BPA exposure effects on spontaneous adipogenesis of the *3T3L1* preadipocytes. *3T3L1* cells were pretreated at 1 nM BPA or vehicle for 8 days. Adipocyte differentiation was induced at D0 without the addition of adipocyte differentiation mix. BPA or vehicle was added during the pretreatment and differentiation process. (**A**,**B**) *Pparγ* mRNA levels were assessed during adipocyte differentiation by qPCR. Results are the mean ± SD (*n* = 4). (**C**) The relative mRNA levels of *Cebpα*, *Ap2*, and *Glut4* were determined at D8 of the differentiation process by qPCR. Results are the mean ± SD (*n* = 4). (**D**) Quantification of Oil Red O staining of *3T3L1* cells stained at D8 of adipocyte differentiation. Results are the mean ± SD (*n* = 3). Statistical significance was tested by the two-tailed Student’s *t*-test. Significant *p* values are indicated as * *p* < 0.05.

To bypass the confounding effect induced by the differentiation mix on promoter methylation, we induced the spontaneous differentiation of *3T3L1* cells without adding the differentiation mix. mRNA was collected at D0, D4, and D8. As shown in Figure 3A, despite some degree of variability in the experiments, there was a significant increase in the expression of *Pparγ* at D4 in BPA treated cells (1.2-fold increase, *p* < 0.05), as it happened in the presence of the differentiation mix. However, upon analyzing the data as relative fold change at D0 (Figure 3B), the *Pparγ* expression at the end of differentiation was dramatically reduced compared to levels reached in the presence of the differentiation mix (Figure 2C), both in the exposed and unexposed cells. We also evaluated (Figure 3C) *Cebpα*, *Ap2*, and *Glut4* as marker genes of terminal adipocyte differentiation, and their expression did not show any significant difference between BPA exposed and control cells. Adipocyte differentiation of *3T3L1* cells was also assessed by evaluating the lipid content by Oil Red O staining at D8 of differentiation (Figure 3D) and no differences were detected in lipid accumulation between exposed and control cells. Thus, *Pparγ* expression and demethylation was also induced in the absence of differentiation mix in BPA exposed cells; nevertheless, overexpression was unable to complete the differentiation process and make the *3T3L1* cells an in vitro model of spontaneous adipogenesis.

### 3.4. BPA Effects on Non-Adipogenic NIH3T3 Cells. Evaluation of Commitment Phase

Although BPA did not show a clear effect on adipocyte differentiation, we tested its action on the adipocyte commitment phase in non-adipogenic *NIH3T3* cells. We compared the expression and methylation levels of the *Pparγ* gene between *3T3L1* and *NIH3T3* cells before and after the exposure.

**Figure 4:**
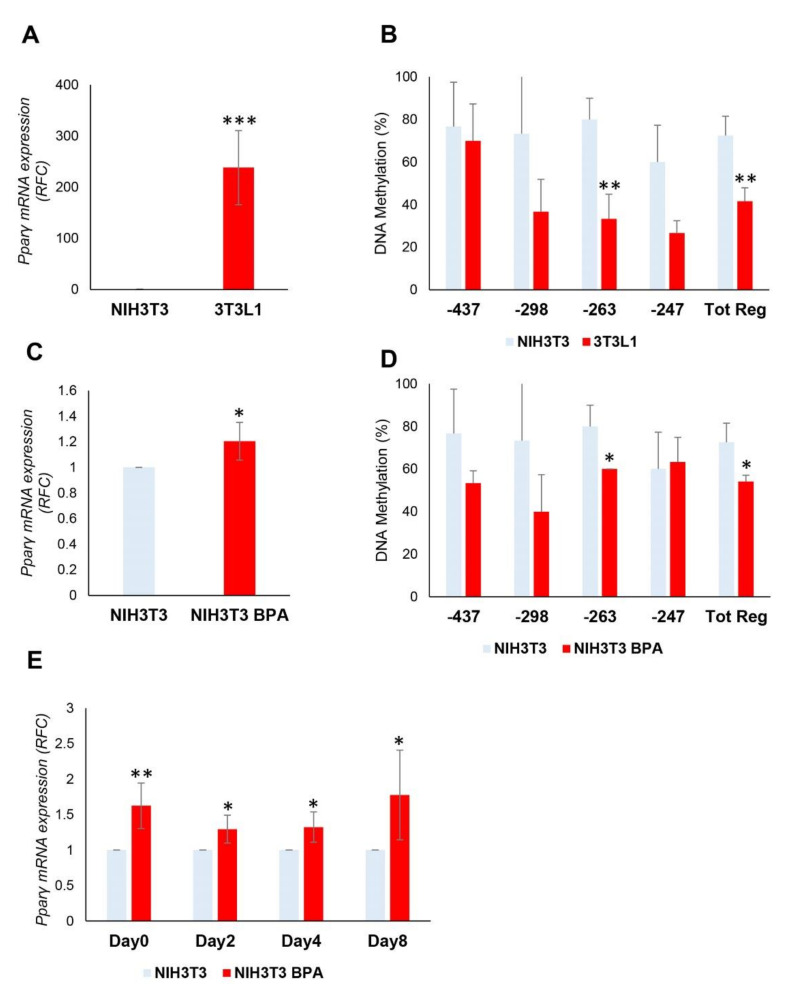

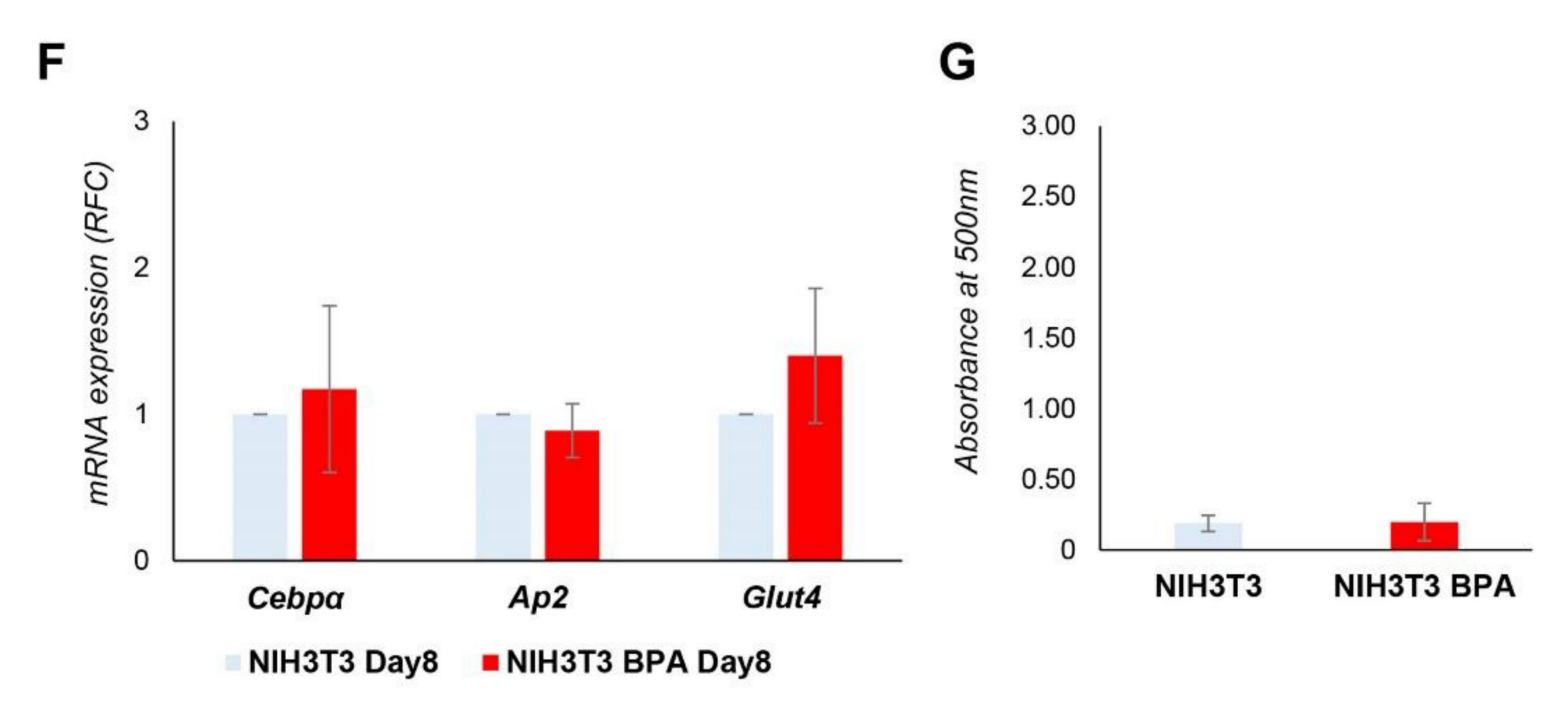
BPA exposure of the non-adipogenic *NIH3T3* fibroblasts. (**A**,**B**) The mRNA expression levels and DNA methylation profile of the *Pparγ* gene were evaluated in *3T3L1* and *NIH3T3* cells (*n* = 3). (**C**) *NIH3T3* cells were pretreated at 1 nM BPA or vehicle for 8 days. *Pparγ* mRNA levels were assessed by qPCR. Results are the mean ± SD (*n* = 5). (**D**) Bisulfite sequencing analysis for DNA methylation assessment of the *Pparγ* promoter region in *NIH3T3* cells exposed or not to BPA. Results are the mean ± SD (*n* = 3); for each experiment, at least ten clones were analyzed by Sanger sequencing. (**E**) *NIH3T3* cells were pretreated at 1 nM BPA or vehicle for 8 days. Adipocyte differentiation was induced at D0 through the administration of a specific adipogenic mix. Cells were pretreated and differentiated with BPA or vehicle. *Pparγ* mRNA levels were assessed during adipocyte differentiation process by qPCR. Results are the mean ± SD (*n* = 4). (**F**) The relative mRNA levels of *Cebpα*, *Ap2*, and *Glut4* were determined at D8 of the differentiation process by qPCR. Results are the mean ± SD (*n* = 3). (**G**) Quantification of Oil Red O staining of *NIH3T3* cells stained at D8 of adipocyte differentiation. Results are the mean ± SD (*n* = 3). Statistical significance was tested by the two-tailed Student’s *t*-test. Significant *p* values are indicated as *** *p* < 0.001, ** *p* < 0.01 and * *p* < 0.05.

Before the exposure, the *Pparγ* mRNA expression (Figure 4A) was significantly increased in *3T3L1* compared to *NIH3T3* (238-fold increase, *p* < 0.001), in which the expression was barely detectable. The contribution of DNA methylation to the transcriptional regulation of *Pparγ* gene was also examined. The DNA methylation of *Pparγ* promoter in *NIH3T3* cells (Tot. Reg. CpG methylation %: 72.5 (*NIH3T3*) versus 41.7 (*3T3L1*), *p* < 0.01) (Figure 4B) was much higher than in *3T3L1* cells, thus reflecting the mRNA expression levels. We also found no sequence variation at the *Pparγ* promoter in *NIH3T3* and *3T3L1* cells (data not shown), suggesting that the differential expression observed may be attributed to the different epigenetic profile.

Successively, *NIH3T3* cells have been treated at a low dose of BPA (1 nM) for 8 days to assess whether BPA also regulates *Pparγ* DNA methylation in non-adipogenic *NIH3T3* cells. As shown in Figure 4C, the mRNA expression of *Pparγ* was significantly increased by BPA treatment in *NIH3T3* fibroblasts (1.2-fold increase, *p* < 0.05). We also tested the ability of BPA to remove the transcriptional block imposed on the *Pparγ* gene in the *NIH3T3* cells. Even in *NIH3T3* cells, the DNA methylation of the Tot. Reg. of the *Pparγ* promoter (Figure 4D) was reduced by BPA exposure (CpG methylation %: 72.5 (*NIH3T3*) versus 54.2 (*NIH3T3* BPA), *p* < 0.05).

After 8 days of BPA pretreatment, adipocyte differentiation was induced in *NIH3T3* cells with the differentiation mix to test the BPA effect on the adipocyte commitment phase. As shown in Figure 4E, BPA exposure significantly increased *Pparγ* expression at D0 (1.6-fold increase, *p* < 0.01), D2 (1.3-fold increase, *p* < 0.05), D4 (1.3-fold increase, *p* < 0.05), and D8 (1.8-fold increase, *p* < 0.05) of differentiation. While no differences in mRNA expression have been detected when we evaluated other marker genes of terminal adipocytes differentiation at D8 (Figure 4F), such as *Cebpα*, *Ap2*, and *Glut4*. Adipocyte differentiation of *NIH3T3* cells was also assessed by evaluating the lipid content by Oil Red O staining at D8 of differentiation (Figure 4G).

The increase in *Pparγ* gene expression during and at the end of the differentiation process (Figure 4E) was more evident in *NIH3T3* compared to *3T3L1* cells (Figure 2B). The hypermethylation profile of the *Pparγ* promoter in *NIH3T3* cells makes the transcriptional effects of BPA stronger. However, BPA exposure does not increase the ability of the *NIH3T3* cells to differentiate in mature adipocytes.

### 3.5. BPA-Induced Alterations Are Reversible after the Termination of the Exposure

**Figure 5:**
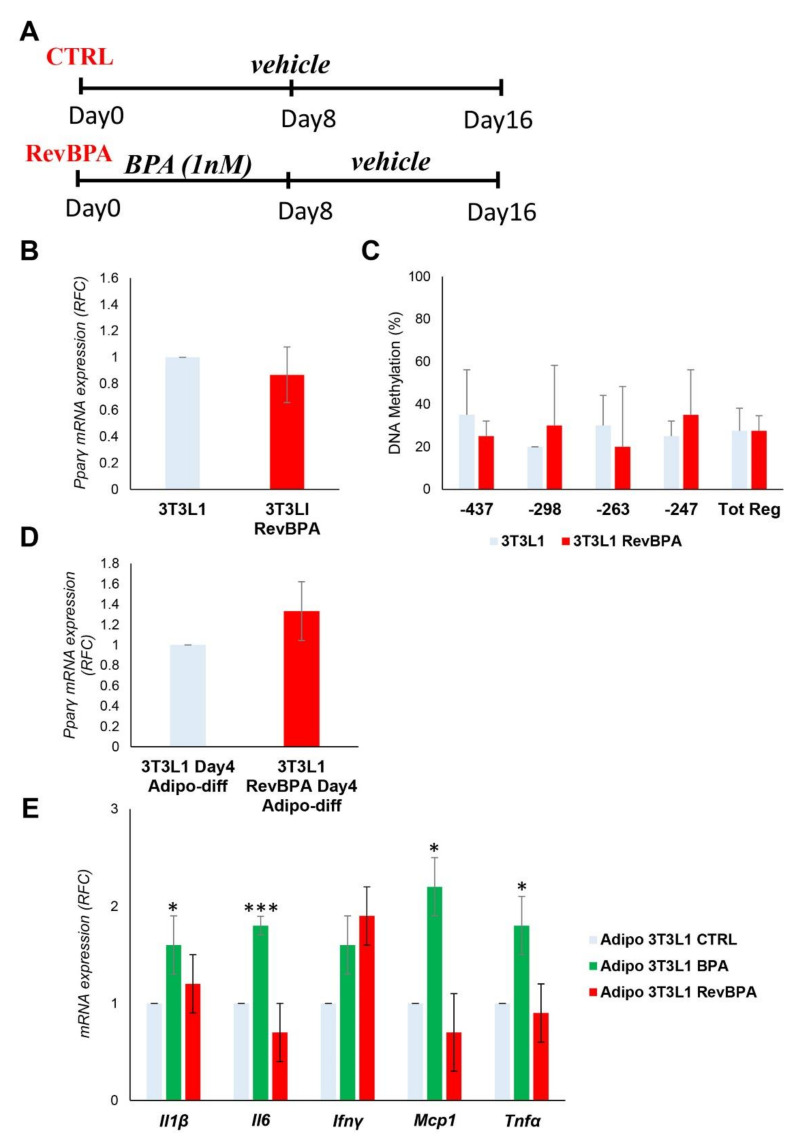
Effects of BPA exposure termination in *3T3L1* cells. (**A**) A schematic representation showing the experimental design. *3T3L1* cells were pretreated at 1 nM BPA or vehicle for 8 days, then the medium was replaced without BPA for 8 days more. Control cells were grown with vehicle for 16 days. (**B**) The mRNA expression levels of the *Pparγ* gene were evaluated by qPCR after termination of the BPA exposure (RevBPA). Results are the mean ± SD (*n* = 3). (**C**) Bisulfite sequencing analysis for DNA methylation assessment of the *Pparγ* promoter region in *3T3L1* cells after termination of the BPA exposure (RevBPA). Results are the mean ± SD (*n* = 3); for each experiment, at least ten clones were analyzed by Sanger sequencing (**D**,**E**) After 16 days, adipocyte differentiation was induced in both control and pre-exposed cells. (**D**) *Pparγ* mRNA levels were assessed by qPCR at D4 of adipocyte differentiation. Results are the mean ± SD (*n* = 3). (**E**) The relative mRNA levels of the proinflammatory cytokines *Il6*, *Ifnγ*, *Tnfα*, *Mcp1*, and *Il1β* were determined at D8 of the adipocyte differentiation process by qPCR. Results are the mean ± SD (*n* = 3). Statistical significance was tested by the two-tailed Student’s *t*-test. Significant *p* values are indicated as *** *p* < 0.001 and * *p* < 0.05.

Following the identification of the epigenetic modification at the *Pparγ* promoter, we tested whether it was stable over time after the termination of the exposure. After BPA exposure for 8 days of the *3T3L1* cells, the medium was replaced with the standard growth medium without BPA for another 8 days (Figure 5A). From now on, we will refer to this specific experimental condition as revBPA. Control cells were grown only with vehicle for 16 days. Both DNA and mRNA were collected at the end of the experiment. As reported in Figure 5B, the mRNA expression of *Pparγ* in preadipocytes was not modified by termination of BPA exposure. To evaluate the memory of the epigenetic trait, we evaluated the methylation level of the *Pparγ* promoter by sodium bisulfite sequencing. As shown in Figure 5C, returning *3T3L1* cells to standard growth conditions was accompanied by a restoration of DNA methylation to values comparable to those measured in control cells, grown with the standard culture medium through the entire 16-day period (Tot. Reg. CpG methylation %: 27.5% (*3T3L1*) versus 27.5% (*3T3L1* revBPA)). We also induced adipocyte differentiation in switched experiment and we confirmed a normalization of the *Pparγ* expression at D4 of the differentiation process (Figure 5D). These data show that, in our experimental conditions, BPA-induced epigenetic modification is reversible and termination of the exposure restores the normal epigenetic profile.

Furthermore, we confirmed that BPA affects the expression of specific cytokines and inflammatory factors in our experimental conditions (Figure 5E). Nevertheless, we examined whether the termination of BPA exposure could not only restore the normal epigenetic pattern of *Pparγ* promoter, but also the expression profile of the proinflammatory cytokines interleukin 6 (*Il6*), interferon γ (*Ifnγ*), tumor necrosis factor α (*Tnfα*), monocyte chemoattractant protein 1 (*Mcp1*), and interleukin 1β (*Il1β*). After stopping the exposure, adipocyte differentiation was induced in both control and pre-exposed cells. As shown in Figure 5E, the termination of BPA exposure was accompanied by a reduction in the expression of all inflammatory cytokines.

The termination of BPA exposure restores not only the normal epigenetic pattern at the *Pparγ* locus and its mRNA expression during the adipocyte differentiation, but also the expression profile of proinflammatory cytokines. Such findings lead us to suggest that exposure to BPA, even at a low dose, induces adipocyte dysfunction, in terms of alteration of specific epigenetic profiles and secretion of proinflammatory cytokines. However, the termination of the exposure is able to revert the expression profile of proinflammatory cytokines induced by the BPA.

## 4. Discussion

We and others have previously demonstrated that exposure to low doses of BPA during adipocyte differentiation results in dysfunctional adipocytes characterized by decreased insulin-stimulated glucose uptake and increased proinflammatory cytokines expression such as *TNFα* and *IL6* [74,77,78]. Therefore, based on the evidence in the literature, it is generally accepted that chronic BPA exposure may generate a low-grade inflammatory state, which affects tissue sensitivity to insulin. In this study, we investigated the effects of BPA on adipogenesis and the molecular mechanisms of its action. In detail, we tested the effects of BPA on the different phases of adipogenesis. Schematically, adipogenesis mainly consists of a commitment phase and a terminal differentiation phase. To address the issue, we used two cell lines: the *3T3L1* and the *NIH3T3*, which are respectively committed and uncommitted towards the adipocyte lineage. The cells were chronically exposed to low concentrations of BPA to mimic human exposure [24,26,36,53,78,79,80]. BPA follows a non-monotonic dose-response curve; therefore, even low concentrations of the contaminant cause biologically relevant and potentially different effects from high concentrations [81,82,83]. Compared to other published papers, the strength of our study is represented by the detailed investigation of all the adipogenesis phases, which take into account both the commitment and terminal differentiation phases. To the best of our knowledge, BPA effect on *NIH3T3* fibroblasts, not committed to the adipocyte lineage, has never been verified before. The identification of the effects exerted by BPA on DNA methylation represents another important novelty of our study.

We provided evidence that low-dose BPA reduces DNA methylation at the *Pparγ* promoter, without affecting mRNA expression in adipose precursor cells. The promoter region of the *Pparγ* gene between −37 and −247 bps, where methylation levels have been tested, is an important regulatory region [68,84]. It includes two Cebpα binding sites (a key regulator of *Pparγ* expression), [85] and therefore, its demethylation is important during the adipocyte differentiation [84]. Since the expression levels of *Cebpα* are almost undetectable in preadipocytes, it is understandable why the demethylation of the *Pparγ* promoter is not paralleled by the increase in its expression in precursor cells. Demethylation of the *Pparγ* promoter leads to the release of the transcriptional inhibitor methyl CpG-binding protein 2, creating a favorable environment for the induction of gene expression. However, further chromatin remodeling, via the binding of the nucleosome remodeling complex, is required for the increase in gene expression [86]. Among the environmental pollutants, only BDE-47 (2, 2′, 4, 4′-tetrabromodiphenyl ether) has been identified as being able to induce *Pparγ2* promoter demethylation accompanied by disruption of glucose homeostasis [68].

Among the epigenetic changes analyzed, DNA methylation seems to be the only responsible for the BPA action. Indeed, a bioinformatics analysis carried out using two different prediction algorithms (miRDB and TargetScan) allowed us to identify about 27 microRNAs that have *Pparγ* as their target gene. Among these, we searched those microRNAs whose expression can be affected by exposure to BPA [87,88,89,90,91]. Following this intersection, two candidate microRNAs, microRNA 27a and 27b, were selected from the above-mentioned list. However, in our experimental conditions, the expression of microRNA 27a and 27b are not modulated by BPA exposure. This does not exclude that, in other cellular models and at different BPA concentrations, these or some other microRNAs may be altered.

Interestingly, in differentiating *3T3L1* cells, we showed a transient increase in *Pparγ* expression and lipid accumulation at D4 of differentiation. The increase in *Pparγ* expression is accompanied by its promoter demethylation. Our hypothesis is that BPA may act as an early initiator of *Pparγ* upregulation, which then occurs during the differentiation. In fact, it induces the demethylation at the *Pparγ* locus, making its promoter predisposed to transcription factor binding (whose expression is induced by the differentiation mix) capable of promoting its expression earlier than in the control cells. We detected a transient increase of the *Pparγ* expression with increased lipid accumulation at D4 in BPA exposed cells. However, this was no longer significant at D8. This is likely due to the complete demethylation of the *Pparγ* promoter as a differentiation mix effect. This could be the reason why discrepancies exist in the literature regarding *Pparγ* regulation and lipid accumulation in *3T3L1* cells exposed to BPA during the adipocyte differentiation. The transient increase in *Pparγ* expression is in agreement with the study by Ohlstein et al., conducted in human adipose stem cells [63]. In this study, we identify the molecular mechanism responsible for the early induction of *Pparγ* expression in BPA exposed cells during the adipocyte differentiation.

Both BPA and differentiation mix reduce *Pparγ* promoter methylation [84]; it is not surprising that BPA anticipates the *Pparγ* overexpression that normally occurs during differentiation. During the adipocyte differentiation, an open chromatin structure prone to be transcribed is obtained at the *Pparγ* locus in both the control and BPA exposed cells, but at different time points. The open chromatin conformation is not sufficient to keep the *Pparγ* gene overexpressed beyond the D4 of differentiation in BPA exposed cells. DNA methylation is only the first step involved in the *Pparγ* regulation, but further chromatin rearrangements are required for the complete gene induction [86]. The transient increase in lipid accumulation and *Pparγ* overexpression between D4 and D8 could be recognized as an enhancement of the adipogenic ability of precursor cells. Furthermore, the expression levels of the other adipogenic marker genes confirm an identical ability of adipocyte differentiation in control and exposed cells. Nevertheless, we cannot exclude that BPA could have a different impact on the adipogenesis at different concentrations and/or exposure time.

To discriminate the effect of BPA from that exerted by the components of the differentiation mix, we differentiated the *3T3L1* cells without the differentiation mix. Both the differentiation mix and BPA induce demethylation of the *Pparγ* promoter [84,86]. Even in the absence of the differentiation mix, BPA induced a significant increase in *Pparγ* expression at D4 of adipocyte differentiation. BPA acts as the initiator of its overexpression, even in the absence of the differentiation mix. However, the slight increase of *Pparγ* expression at D8 compared to D0 in the absence of mix does not allow to consider *3T3L1* as an in vitro model of spontaneous adipogenesis when exposed to BPA. To drive the differentiation process of the *3T3L1* cells, it is necessary to recruit a transcriptional apparatus (C/EBPs proteins family) to the *Pparγ* promoter, induced by the administration of the differentiation mix. As already published, the overexpression of the only Pparγ transcription factor could not compensate for the absence of the differentiation mix [92], even in BPA exposed cells. The wide variability observed in this part of the results is due to the long experimental condition setting of the spontaneous adipocyte differentiation.

To examine the effects of BPA on the commitment phase, we used uncommitted fibroblast *NIH3T3* cells. In the *NIH3T3* compared to the *3T3L1* cells, the expression of *Pparγ* is barely detectable and its promoter is completely methylated. The transcriptional effect of BPA in *NIH3T3* cells is clearer than in *3T3L1* cells; indeed, *Pparγ* is overexpressed in preadipocytes and during the differentiation protocol. Interestingly, BPA, at the same concentration, has a different transcriptional effect depending on the cell type (exposed). The *Pparγ* promoter is completely methylated in the *NIH3T3* cells, and this makes the overexpression more stable. Despite increased expression levels, *NIH3T3* cannot fully differentiate in mature adipocytes. Although *Pparγ* is the key gene in the differentiation process, it cannot sustain the complete transformation into mature adipocytes, as previously reported by others [92]. Experiments of C/EBP protein family overexpression and chronic BPA exposure could provide evidence to support this hypothesis. However, the main goal of our study was not to transform uncommitted fibroblasts into committed preadipocytes, and accordingly, we will look at this in the future.

On the other hand, understanding whether the detected changes induced by BPA are reversible, by blocking BPA exposure, will have an important impact on human health and the replacement policies of this component in industrial use. Persistence of certain epigenetic marks keeps memory of exposure to environmental hits [93]. Therefore, we stopped BPA exposure for an additional 8 days and we verified the stability of the previously identified BPA-induced alterations. These experiments proved that both the methylation of the promoter and the *Pparγ* expression at D4 of differentiation are completely restored at the level found in the control cells. This underlines how these epigenetic marks are not stable and can be reverted following exposure termination. We hypothesize that the enzyme machinery, capable of writing/erasing these epigenetic changes, is reversibly activated after BPA exposure. However, further investigations are needed to support our hypothesis. Proving the reversibility of these changes highlights the importance of all policies aimed at blocking human exposure to this contaminant. Therefore, the epigenetic modification induced by BPA and its reversibility constitutes an important novelty of our study.

Another effect of BPA-induced adipocyte dysfunction is represented by the increased production of proinflammatory cytokines [24,53,58,74,77,78]. It is widely documented that a local inflammatory state of adipose tissue (particularly of the SAT) is associated with the establishment of local insulin resistance. Through amplification mechanisms, also involving immune cells, local insulin resistance worsens to a more severe state, which is systemic insulin resistance [59,60,75]. This observation, together with the previously published studies in animal models [25,94,95,96], suggests that BPA may be a more potent diabetogenic agent than an obesogenic one. Interestingly, our findings have also shown that blocking BPA exposure is not associated with a memory of the inflammatory state. The transcription of inflammatory cytokines directly involved in the onset of insulin resistance (*Il1β*, *Il6*, and *Tnfα*) and chemotactic factors for macrophage recruitment (*Mcp1*) are restored to comparable levels in the cells never exposed to the pollutant. This evidence also has important insights into the prevention of metabolic complications associated with BPA exposure.

Our study has potential limitations. Although replicated in two different cell lines, our results on BPA-mediated effects were obtained in in vitro systems of murine preadipocytes. It will be interesting to confirm these observations in vivo in a mouse model exposed to BPA, which will allow to reveal the systemic effect of BPA exposure. Furthermore, this study lacks human preadipocyte samples. We plan to translate these findings in humans, using preadipocytes isolated from the stromal vascular fraction of SAT biopsies. However, in this first part of the study, the advantages offered by an in vitro system (ability to expose cells directly to chemical, ability to manipulate environmental conditions, and evaluation of intrinsic cell response to chemical) directed us towards this experimental design.

## 5. Conclusions

We identified the DNA methylation as an alternative transcriptional mechanism by which BPA affects gene expression. *Pparγ* could represent only the first identified target gene, and other genes, including inflammatory cytokines, can be regulated by shared epigenetic mechanisms [97,98,99,100]. At these low concentrations, the effects induced by BPA do not alter the adipogenesis mechanism. However, the DNA methylation effect of the BPA is clear and replicated in all cell lines and experimental conditions used. The effects on mature adipose tissue remain to be investigated. All our data show how the intervention policies aimed at replacing this pollutant and reducing individual exposure to BPA have been taken appropriately. Exposure to low doses BPA causes reversible effects; stopping the exposure and the induction of inflammatory cytokines can prevent the progression towards more serious metabolic complications.

## Figures and Tables

**Table 1:** A complete list of primers used in this study.

Gene name	Primer sequence (5′ to 3′)
*CCAAT/enhancer binding protein alpha (Cebpα)*	Forward: CGCGAGCCAGTTGGGGCACT Reverse: GGGGCTCTGGAGGTGACTGCT
*Cyclophilin A*	Forward: GCAGACAAAGTTCCAAAGACAG Reverse: CACCCTGGCACATGAATCC
*Fatty acid binding protein 4 (Fabp4 or Ap2)*	Forward: TCTCACCTGGAAGACAGCTCC Reverse: GCTGATGATCATGTTGGGCTTGG
*Glucose transporter member 4 (Glut4)*	Forward: CAATGTCTTGGCCGTGTTGG Reverse: GCCCTGATGTTAGCCCTGAG
*Interferon γ (Ifnγ)*	Forward: CCAAGCGGCTGACTGAACTC Reverse: CACTGCAGCTCTGAATGTTTCTT
*Interleukin 1β (Il1β)*	Forward: AGAGCCTGTGTTTCCTCCTTG Reverse: AAAGACCTCAAGTGCAAGGCTA
*Interleukin 6 (Il6)*	Forward: GGAGTGGCTAAGGACCAAGAC Reverse: GCATAACGCACTAGGTTTGCC
*Monocyte chemoattractant protein 1 (Mcp1)*	Forward: CTGTAGTTTTTGTCACCAAGCTCA Reverse: GTGCTGAAGACCTTAGGGCA
*Peroxisome proliferator-activated receptor gamma 2 (Pparγ2)*	Forward: CAGTGGAGACCGCCCAGGCT Reverse: TGGAGCAGGGGGTGAAGGCT
*Tumor necrosis factor α (Tnfα)*	Forward: AGCCCCGAGTCTGTATCCTT Reverse: CTCCCTTTGCAGAACTCAGG
Primers used for DNA methylation analysis	
*Pparγ2 gene promoter*	Forward: GATGTGTGATTAGGAGTTTTAATTAAA Reverse:CAAACCTAAATTAACTAACACTATCCTAAC

## Supplementary Materials

The following are available online at https://www.mdpi.com/2072-6643/12/11/3498/s1, Figure S1: A schematic flowchart of the experimental design and treatments, Figure S2: Cell morphology and cell viability of the *3T3L1* exposed to BPA, Figure S3: BPA exposure effects on DNA methylation of the *Pparγ* promoter in *3T3L1* Preadipocytes, Table S1: A list of 27 predicted microRNAs targeting the 3’-UTR of *Pparγ* gene obtained from the miRDB and Targetscan online databases.
