# Supplementary material for: Low-dose Bisphenol-A Promotes Epigenetic Changes at Pparγ Promoter in Adipose Precursor Cells
Source: Nutrients. 2020 Nov 13;12(11):3498. doi: 10.3390/nu12113498 (PMC7696502; doi:10.3390/nu12113498)
Supplement: Supplementary file 1 [file nutrients-12-03498-s001.pdf]

Supplementary Figures and Tables

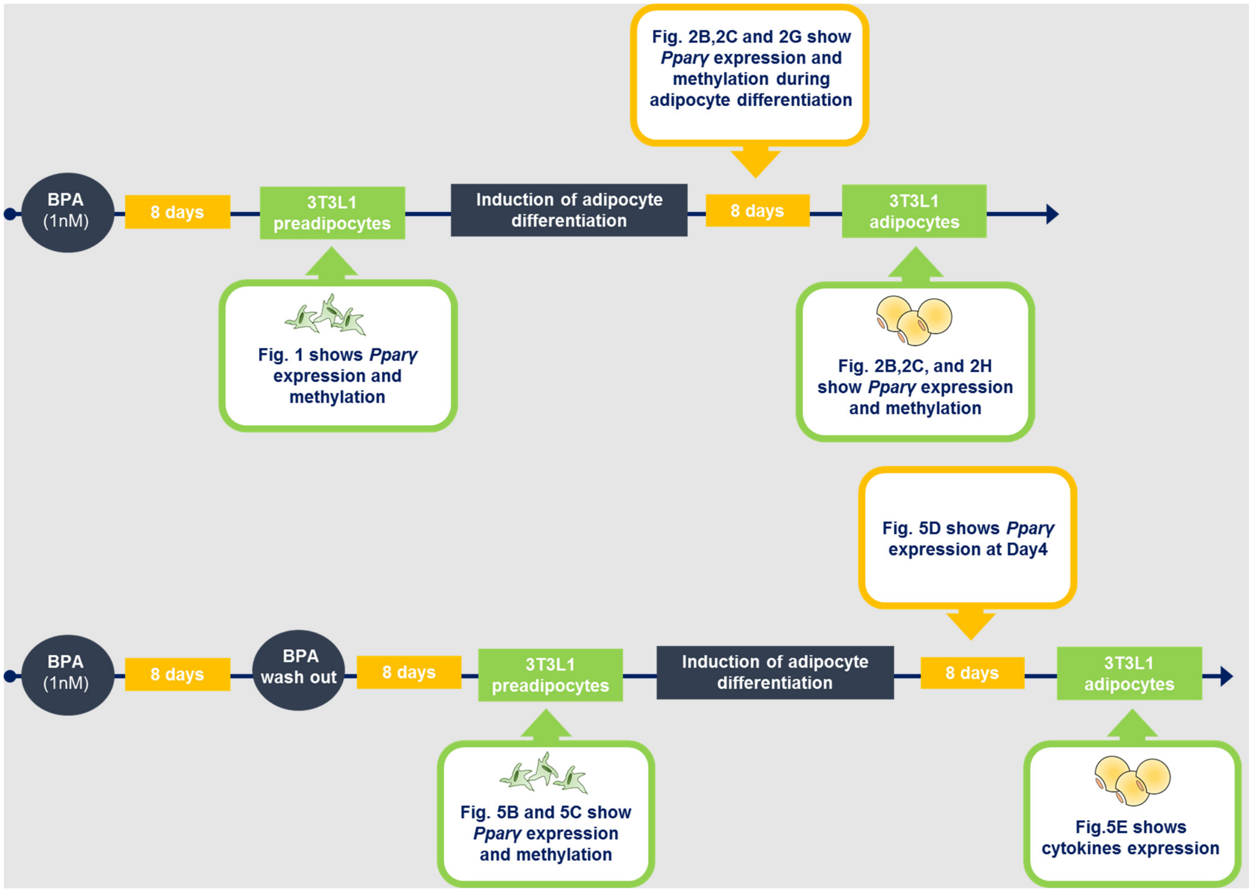

Figure S1. A schematic flowchart of the experimental design and treatments.

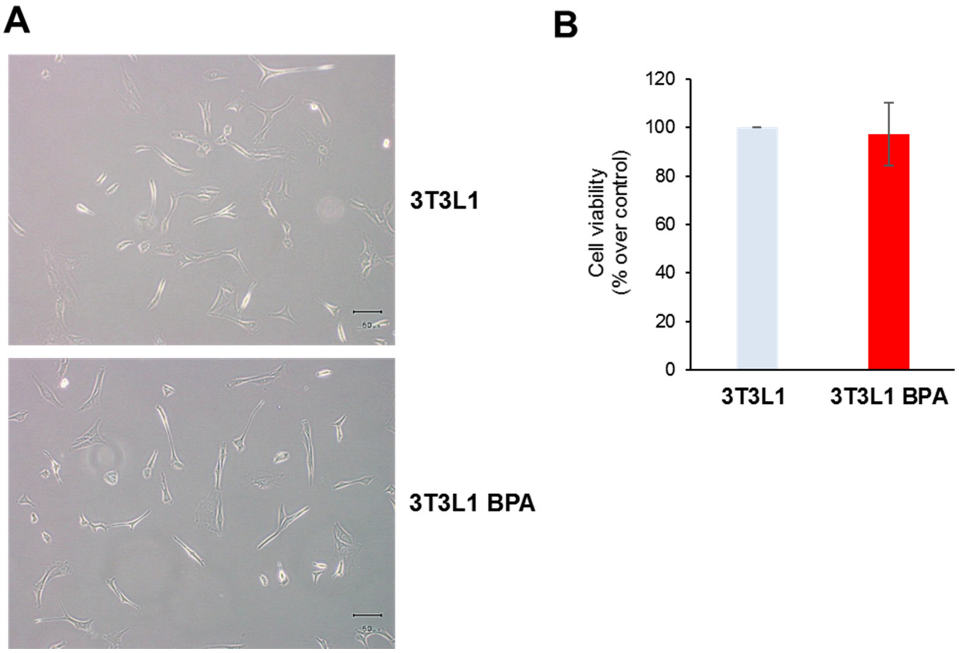

Figure S2. Cell morphology and cell viability of the *3T3L1* exposed to BPA. (**A**) Representative microphotographs of the *3T3L1* cells pretreated with 1 nM BPA or vehicle for 8 days. 10X magnification; scale bars, 50µm. (**B**) Cell viability was assessed by the MTT assay. The percentage of survival was calculated as the absorbance ratio of treated to untreated cells. The data presented are the mean ± SD from three replicate wells, replicated three times.

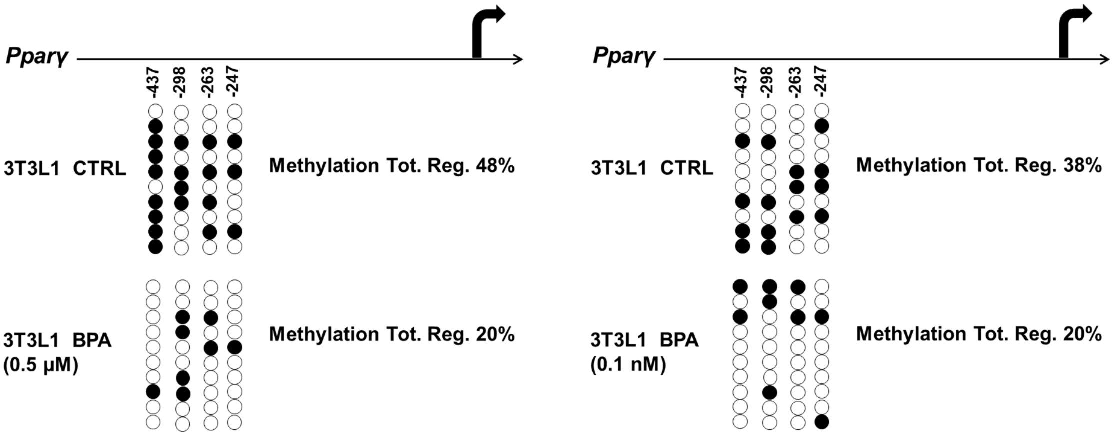

Figure S3. BPA exposure effects on DNA methylation of the *Pparγ* promoter in *3T3L1* preadipocytes. Bisulfite sequencing analysis for DNA methylation assessment of the *Pparγ* promoter in *3T3L1* cells exposed to BPA (0.5 µM and 0.1 nM).

Table S1. A list of 27 predicted microRNAs targeting the 3'-UTR of *Pparγ* gene obtained from the miRDB and Targetscan online databeses.

**microRNAs Targeting *Pparγ G*ene**
mmu-miR-27b-3p, mmu-miR-669k-3p, mmu-miR-27a-3p, mmu-miR-669h-3p, mmu-miR-130c, mmu-miR-130a-3p, mmu-miR-6341, mmu-miR-301b-3p, mmu-miR-301a-3p mmu-miR-130b-3p, mmu-miR-721, mmu-miR-6389, mmu-miR-6986-3p, mmu-miR-7236-5p, mmu-miR-5624-5p, mmu-miR-3473g, mmu-miR-673-5p, mmu-miR-6985-3p, mmu-miR-128-3p, mmu-miR-340-5p, mmu-miR-6539, mmu-miR-590-3p, mmu-miR-338-5p, mmu-miR-335-3p, mmu-miR-6951-3p, mmu-miR-7116-3p, mmu-miR-760-5p
